# The Significance of Dual-Energy X-ray Absorptiometry (DXA) Examination in Cushing’s Syndrome—A Systematic Review

**DOI:** 10.3390/diagnostics13091576

**Published:** 2023-04-28

**Authors:** Aleksandra Radecka, Anna Lubkowska

**Affiliations:** Department of Functional Diagnostics and Physical Medicine, Pomeranian Medical University in Szczecin, Żołnierska 54, 71-210 Szczecin, Poland

**Keywords:** dual-energy X-ray absorptiometry, Cushing’s syndrome, BMD—bone mineral density, VFA—vertebral fracture assessment, ALMI—appendicular lean mass index, FMI—fat mass index, AG—android–gynoid ratio, VAT visceral fat

## Abstract

In recent years, the usefulness of dual-energy X-ray absorptiometry (DXA) as a valuable complementary method of assessing the content and distribution of adipose and lean tissue as well as bone mineral density and estimating the risk of fractures has been increasingly confirmed. The diagnosis and treatment of Cushing’s syndrome remain challenging, and monitoring the effects of treatment is often necessary. DXA tests offer a potential solution to many problems related to the availability of a quick, detailed, and reliable analysis of changes in the content and distribution of individual body composition components. The article discusses total body DXA scans (FMI, VAT, ALMI), lumbar spine scans (VFA, TBS), and osteoporosis scans (BMD, T-score, Z-score)—all are of potential interest in Cushing’s syndrome. The article discusses the use of the most important indicators obtained from a DXA test (FMI, VAT, ALMI, BMD, T-score, Z-score, VFA, TBS) and their clinical significance in Cushing’s syndrome was verified. The literature from the last decade was used for the study, available in MEDLINE, Web of Science, and ScienceDirect.

## 1. Introduction

DXA body composition (BC) is the quantification and pattern of tissues with different X-ray absorption peaks, which with DXA are designated for bone and soft tissue, with the further partitioning of soft tissue into fat and lean compartments. Based on the physics of absorptiometry, DXA measures the mass of the compartments, for the total body area or for sub-regions scanned, and is usually reported as a direct mass measurement for the total body and as areal densities for fracture risk assessment. Body composition (BC) is the quantification of the number and arrangement of the individual elements that make up the body in terms of surface area or sum [1,2]. BC represents the effects of all external and internal factors (genetic, environmental) on the physiology of the body, representing a type of “historical” record of energy balance and nutrient intake [2]. Given the above, the detailed and reliable characterisation of human body composition is now becoming an increasingly popular research problem, especially in the area of metabolic disorders. The optimal method for assessing body composition should be safe, accurate, have high reproducibility, be easy and quick to measure, be affordable, and allow the study of entire populations. Currently, it is not possible to directly measure body components in vivo, and consequently, indirect methods and models have been developed for this purpose. Examples of body composition assessment methods include bioelectrical impedance (BIA), dual-energy X-ray absorptiometry (DXA), computed tomography body composition (CTBC), quantitative computed tomography (QCT), magnetic resonance imaging (MRI), quantitative magnetic resonance (QMR), nuclear magnetic resonance (NMR), and positron emission tomography (PET) [3,4,5,6].

The dual-energy X-ray absorptiometry (DXA) method allows a relatively detailed assessment of the three most important body components, i.e., adipose tissue, lean tissue, and bone tissue. The DXA method is based on the phenomenon of the attenuation of X-rays by tissues. When scanning the body, the X-ray generator emits alternating voltage and double energy pulses. The beam energy value depends on the parameters of a densitometer, which can be, for example, 70 and 140 kV. The effective value of a radiation dose is low, especially in modern densitometers. The effective radiation dose (RED) also depends on the DXA scanning method. It has the lowest values for fast-array scanning, intermediate values for high-resolution (HD), scanning and the highest values for array scanning. The radiation energy is attenuated in different ways (absorbed or diffused) by anatomical structures, depending on the intensity of energy emitted by a generator and the density and thickness of tissue [6,7]. Thanks to the daily calibration with phantoms, the stability of measurement can be controlled, and the variability of the obtained results can be kept below 0.5% for many years [6,8,9]. An important advantage of the DXA method is the low exposure of both the operator and the subject to ionising radiation, which significantly reduces the number of contraindications and increases the safety of examination [7,9,10,11]. DXA testing does not require the active participation of subjects, which is a particular advantage in elderly and dysfunctional patients [3,12]. In estimating body component values, the DXA method has not only gained international acceptance by expert groups but is also indicated as a reference method [13,14,15,16]. Disadvantages of the DXA method include the large size of the device, which prevents its mobility, and the influence of the pathological state of body hydration on the obtained values—for example, in the course of diseases with hypervolemia (heart, kidney or liver failure). A particularly significant disadvantage of the DXA method in determining fat-free indices is that it does not separate intramuscular fat from actual muscle tissue, which is to be kept in mind in measurements taken in highly obese individuals [6,8,10,13,17].

The DXA method is recommended for assessing body composition in clinical, population, cross-sectional, and longitudinal studies in all periods of human life [6,10,11,12,13,15,16,17,18]. The DXA method is considered the gold standard in body composition analysis at the molecular level, providing the quantification and evaluation of total tissue mass, which is partitioned between bone mineral (bone mineral content, BMC) and soft tissue mass, based on respective absorption peaks. In non-bone mineral pixels, the soft tissue is further partitioned into fat (FM) and lean mass (LM) (three-compartment model). The measurement can be applied to a single body area as well as the whole body, allowing the determination of several absolute parameters and relative indices to characterise the distribution of adipose tissue [1,10,12], estimate the compactness of muscle mass and bone mass, and determine its strength (risk of or finding of osteopenia, osteoporosis, fractures) [19]. The DXA method enables the determination of absolute and relative parameters based on the content of lean, adipose, and bone mass. 

In the clinical use of DXA, single body-area scans of the spine, hip, and forearm should be separated from whole-body scans. Based on the WHO criteria for osteoporosis and osteopenia, DXA examination for the hip (posterior-anterior, PA), lumbar spine (LS), and/or the forearm are the most widely used techniques, being the gold standards in diagnosing and monitoring osteoporosis. The primary indicator is bone mineral density (BMD), expressed in g/m^2^, measured in well-defined areas of interest in these images [19,20]. BMD is considered an indicator of high reliability and precision, capable of predicting osteoporotic fractures well in advance and valuable in the follow-up of patients with osteoporosis. The absolute value of BMD is matched to the mean BMD of a healthy adult reference population and, depending on the age of that population, is reported as a T-score or Z-score. The number of standard deviations by which BMD differs from the BMD of the healthy young adult reference population is referred to as the BMD T-score. In contrast, the standard deviation from an individual’s BMD from the mean value of the age- and sex-matched reference population is named the Z-score [21,22,23,24,25]. The introduction in 1993 of fan-beam radiation source technology (associated with multiple detectors) in place of pencil densitometers (emitting a single rectilinear, highly collimated beam of X-rays related to a single detector) made it possible to reduce radiation exposure time while improving the quality of the images obtained. The improvement in the quality of images obtained has enabled the development of new applications of the DXA method in the diagnosis of vertebral compression fractures using vertebral fracture assessment (VFA), referred to as the trabecular bone score (TBS) [26]. VFA (vertebral fracture assessment) is a bone assessment tool to detect fractures, recommended as an alternative to standard radiography by ISCD, with significantly lower radiation (2–60 µSv versus about 600 µSv during radiographs of the thoracic and lumbar spine). VFA is recommended in screening people with a risk of osteoporosis and the follow-up of osteoporotic patients undergoing treatment [27,28,29]. The lumbar spine trabecular bone score (TBS) is a grey-level texture measurement based on experimental variograms previously obtained from the two-dimensional (2D) projection images acquired during the DXA lumbar spine scan. Studies have robustly shown that TBS is an independent predictor of fragility fractures and predicted the fracture risk independently of FRAX BMD and other clinical risk factors for fracture [19,30,31,32,33]. The whole-body scan provides values for indicators such as BMC, BMD, fat-free mass, and fat mass for the whole body and by area and ROI (region of interest) [28]. Although the whole-body scan (WBS) is performed by the same method, it provides accurate values of the scanned components, and it has yet to fully move into the clinical domain despite the efforts of manufacturers and researchers [26]. The main indices determined from WBS used clinically are appendicular lean mass (ALM; the sum of LMs of the upper and lower extremities) [34,35]; appendicular lean mass index (ALMI), which considers body height [16,36]; fat mass index (FMI); android/gynoid ratio (AG); and visceral adipose tissue (VAT) [1]. The ALMI index in research, as well as clinical practice, is used for assessing the muscle mass in the diagnosis and monitoring of sarcopenia in the elderly [16,37]; assessing the active tissue content in children with cerebral palsy [38], athletes [39], and post-menopausal women [40]; determining the relationship of skeletal muscle mass to blood pressure [41]; hyperinsulinaemia [42]; and characterising the body composition of healthy children and adults [43,44]. FMI is now proposed as a measure of obesity (instead of BMI) with cut-off points that have been developed [45]. The AG value of more than 1 refers to an increased risk of dyslipidaemia, insulin resistance, and other cardiovascular risk factors (impaired glucose tolerance, hypercholesterolaemia, hypertriglyceridaemia, and hypertension) [35,46,47]. The clinical importance of visceral adipose tissue measurements stems from its association with the adverse metabolic profile and cardiometabolic risk factors [23], which has also been confirmed for DXA values [48,49]. T-score and Z-score parameters are also calculated for the whole-body scan, but T-score and Z-score for the whole skeleton cannot be used as a basis for diagnosing osteoporosis but only as a screening test for an overall assessment of BMD [26]. Clinical whole-body DXA scanning is used to assess fat redistribution and changes in lean and bone mass among patients after bariatric surgery [50,51], in HIV-infected patients receiving antiretroviral therapy [52,53], and in patients with metabolic disorders (metabolic syndrome [54], Pompe’s disease [55], Cushing’s syndrome [56]), as well as anorexia [57,58] and suspected sarcopenia [59]. In addition, VAT and AG indices are used in assessing the risk of sudden cardiovascular incidents. The latest generation of densitometers allows for the imaging of calcifications in the abdominal aorta (via high-definition (HD) VFA examination on the AAC-8 visual scale) [60,61]. 

The most clinically important parameters are briefly presented and summarised in Table 1. 

Cushing’s syndrome is associated with abnormalities in the muscle and bone structure and function and abnormal fat distribution, which seems to be an essential indication for the clinical use of DXA testing. Cushing’s syndrome (CS) is an eponym that honours the seminal description of endogenous hypercortisolism by Dr Harvey Cushing. The cause of CS is often exogenous (iatrogenic due to pharmacological doses of synthetic glucocorticoids). However, CS can also occur from an endogenous cause, e.g., ACTH (adrenocorticotropic hormone)-dependent, about 80%; Cushing’s disease (CD); ectopic ACTH-secreting tumours; CRH (corticotropin-releasing hormone)-secreting tumours; and ACTH-independent (20%; adrenal tumour, carcinoma and macroscopic overgrowth (AIMAH)). CD is a rare disorder caused by pituitary corticotropic tumours and is the most common cause (nearly 70% in adults) of endogenous CS [67,68,69,70]. Independent of the reason, high plasma glucocorticosteroid concentrations cause several adverse metabolic changes and characteristic clinical symptoms. Cushing syndrome (CS), according to the ICD-11 classification, belongs to the endocrine, nutritional, or metabolic disease group under code 5A70. Its clinical manifestation is referred to as a “model” of a metabolic syndrome, the features of which can persist long after cortisol levels normalise [71]. Hypercortisolaemia affects not only the biology but also the endocrine function of adipose tissue, regulating lipolysis, adipocyte differentiation, and the transcription of specific tissue factors [71]. The main clinical manifestations include visceral obesity, signs of proteolysis, hyperglycaemia, and dyslipidaemia. Weight gain with a characteristic central distribution of adipose tissue is the most common and often initial symptom in patients with CD persisting to the normalisation of cortisol levels. Adipose tissue accumulates in the face (“moon face”), the dorsal–upper chest area (“buffalo hump”), and the supraclavicular area, distinguishing CD from simple obesity. The negative effect of cortisol on protein results in its loss, the atrophy of proximal muscles, and reduced strength. The clinical consequences are fatigue, leg weakness, and even impaired gait and function in daily life. Further characteristics include the presence of wide purple stretch marks and skin that is thin and sensitive to minor injuries, leading to frequent bruising, ulceration, and infection. Hypercortisolism leads to general osteoporosis due to decreased osteoblast function (the result of osteoblast and osteocyte apoptosis and the inhibition of osteoblastogenesis), increased bone resorption, impaired intestinal calcium absorption, and decreased renal tubular calcium reabsorption. These changes mainly affect the trabecular bone of the vertebral bodies, leading to frequent compression fractures [68,69,70]. Chronic excessive cortisol secretion also leads to the development of several comorbidities that include diabetic dyslipidaemia and cardiovascular diseases (arteriosclerosis and hypertension, arterial and venous thrombosis), as well as opportunistic infections, neuropsychiatric disorders (depression, irritability, anxiety and sleep disorders), and cognitive and short-term memory disorders. CS is associated with high mortality, even after successful hypercortisolemia treatment [68,69,70,72]. Despite significant advances, the diagnosis and treatment of CS remain challenging, and monitoring the effects of treatment is often necessary. DXA testing offers a potential solution to many problems associated with the availability of a rapid, detailed, and reliable analysis of changes in the content and distribution of individual body composition components. The more convenient methods (i.e., bioelectrical impedance and skinfold measurements) did not give reliable results.

This review aims to present current applications and selected technical details of DXA testing in Cushing’s syndrome as a condition that intensely affects body composition, mass, and microarchitecture.

## 2. Materials and Methods

### 2.1. Study Design

DXA examination with high accuracy can show changes in bone mineral and bone microarchitecture content, assess bone strength, and predict the risk of bone fractures and also allows for characterising both the content and distribution of fat and non-fat components of the body. It seems that for Cushing’s syndrome, due to its characteristic clinical course characterised by abnormalities in both the content and distribution of all of the above body components, the potential of DXA testing is underestimated and unused. Further, the manner of many metabolic disorders, CS was selected for the following review.

### 2.2. Search Strategies

This review searched for relevant articles in electronic databases, including the National Library of Medicine (MEDLINE), Web of Science, and Science Direct. The search process used the Boolean operator AND/OR in the combination of the following keywords: dual-energy X-ray absorptiometry and Cushing’s syndrome (CS).

### 2.3. Article Protocol Selection

The articles were included for review if they met the following inclusion criteria: (1) published in English with a publication date from 2012 to September 2022; (2) were original studies using DXA to assess body composition in CS; (3) were fully accessed articles, a copy of which the authors could obtain; and (4) were studies involving adult humans. Articles were excluded if they were: (1) older than 10 years; (2) were studies other than clinical trials (meta-analyses, systematic reviews, case reports, expert groups’ recommendation); (3) were papers on DXA or Cushing’s syndrome only (based on the screening of titles and abstracts); (4) were animal studies; or (5) their main results were not in line with the purpose of this literature review. 

At this stage, the work was performed by one researcher (A.R.). Finally, 225 papers were included in the screening of titles and abstracts, which were already independently reviewed by both reviewers (A.R., A.L). Each reviewer selected papers to be rejected and included in the analysis, which was again discussed together this time. Ultimately, 37 articles were included in the detailed screening. After a detailed review, 9 case reports and 10 duplicates were identified and excluded from the analysis. During the selection process, 4 papers inconsistent with the assumptions of the review were excluded (these were expert recommendations, animal studies, and studies involving healthy people who underwent a hydrocortisone infusion and involving people with subclinical hypercortisolism). Ultimately, 14 studies were included in the systematic review.

**Figure 1:**
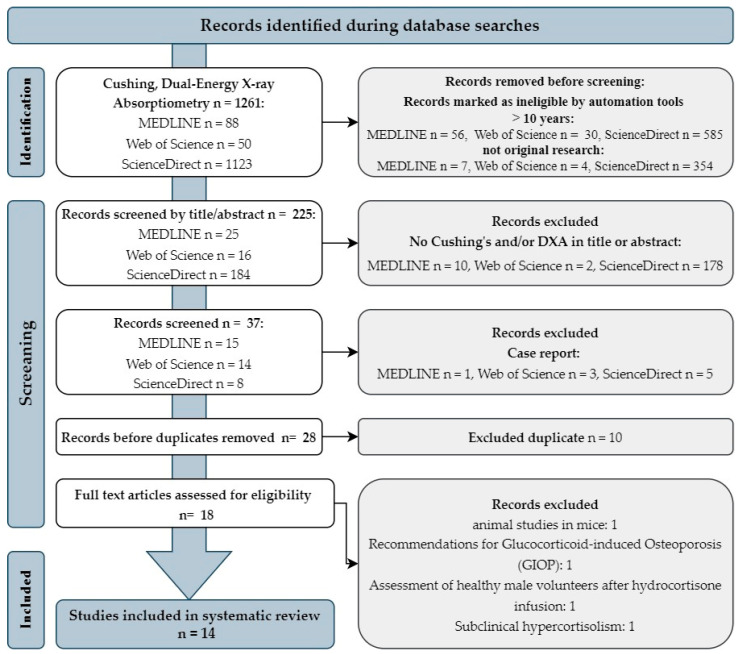
PRISMA (preferred reporting items for systematic reviews and meta-analyses) flow chart.

The complete search process is illustrated in the PRISMA (preferred reporting items for systematic reviews and meta-analyses) flow chart (Figure 1). The search results are presented in the individual subchapters of the manuscript. The studies were grouped for synthesis. The first group of studies focused on the clinical use of the DXA testing of adipose tissue, the second on lean tissue, and the last on bone in Cushing’s syndrome. The summary is provided in Table 2.

Data synthesis and analysis were performed based on a narrative summary of the results of the included studies.

## 3. Importance of DXA Analysis in Patients with Cushing’s Syndrome Based on Clinical Studies

### 3.1. Application of Body Composition Analysis by the DXA Method in Cushing’s Syndrome

#### 3.1.1. Fat Tissue Application

Centripetal obesity with excess VAT develops rapidly in most CS patients. Abnormal body fat content and distribution affect the endocrine function of fat tissue. The excess visceral adipose tissue (VAT) causes low-grade systemic inflammation, resulting in macrophage infiltration in response to microhypoxia and the disruption of adipocytes [85]. The secretion process of adipocytokines by the VAT changes, leading to an adverse adipocytokine profile. It causes insulin resistance, endothelial dysfunction, and, eventually, macrovascular cardiovascular disease, the development of hypertension, diabetes, and an increased risk of cardiovascular events [86,87]. The successful treatment of CS over time results in weight loss and a reduction in waist circumference. However, patients report persistent abdominal fat accumulation [88]. The DXA method used for a detailed analysis of the body composition during treatment and during remission makes it possible to monitor the patient’s condition with particular emphasis on the distribution of fat tissue, constituting an important clinical tool. 

According to Ceccato et al., the application of the DXA method allowed for a detailed characterisation of patients with active CS and then complete follow-up (mean observation time was 32 months) and evaluation during remission in terms of individual fat and non-fat components. DXA evaluation was sufficient to determine whether body composition normalised after the hypercortisolaemia went into remission and whether the effect was dependent on treatment (surgery, pharmacotherapy). The researchers showed that there is a reduction in fat mass during CS remission, which is especially evident after post-operative surgery. A decrease in adipose tissue (mass and percentage) was observed in the whole body, trunk, and R1 locus (−14.4% and −17%, respectively). CS body composition after remission was similar to the matched control group, especially for FM analysed overall in the trunk and R1 box. This change in body composition was accompanied by a decrease in serum glucose levels and a reduction in hypertension and symptoms of metabolic syndrome [73].

Wagenmakers et al., 2015 investigated the adipose tissue distribution and adipocytokine profiles of patients in long-term remission of CS and compared the results with a healthy gender-, age-, and BMI-matched control group. The results obtained by these authors showed that the percentage of total fat and BMI did not differ from healthy controls, but contrary to them, Ceccato et al. found centripetal fat distribution in CS patients in 5-year remission. Persistent central obesity, as demonstrated by DXA, was associated with an unfavourable adipokine profile, with reduced adiponectin levels and elevated resistin and leptin levels, confirming the persistence of an unfavourable metabolic profile despite long-term remission [74].

Similarly, Ragnarsson et al., based on the DXA body composition analysis, confirmed that women with CS in long-term remission have increased abdominal fat mass compared to healthy individuals. The presented results demonstrate that central obesity associated with long-term hypercortisolism in CS is not completely reversible after treatment and anthropometric methods (e.g., BMI) mask it [75].

Stratrova et al. pointed out the phenotypic similarities between obese, metabolic syndrome patients and CS and the possibility of using DXA to determine potential differences in body fat distribution and content in these metabolic disorders. The researchers used a whole-body scan to determine the cut-off point values for central obesity indices, which best differentiate extreme visceral obesity in Cushing’s syndrome from non-CS obese and non-obese women. The authors indicated the COI1 CP value of 0.55 was discovered as a diagnostic criterion of extreme abdominal obesity and COI2 of 0.38 as a diagnostic criterion of normal body fat distribution that excluded abdominal obesity [76].

Continuing the study, Stratrova and her team developed new DXA indices of central abdominal obesity as the ratio of android and torso to legs and torso and legs to total tissue and fat mass, which best differentiate CS. Among many proposed ones, the researchers pointed to A/L-Tm CP of 0.3 and A/L-Fn CP of 0.26 as the best DXA diagnostic indices in extreme abdominal obesity in CS and obese women with MS without CS. They also developed normative values for the index above to exclude abdominal obesity of A/L-Tn CP of 0.23 and A/L-Fn CP of 0.25 [77].

#### 3.1.2. Lean Tissue Application

Active CS is also characterised by reduced lean body mass (LBM), which is directly related to the loss of muscle mass and muscle weakness. It particularly concerns the appendicular muscles of the appendix of the pelvic girdle and lower extremities, regardless of the level of activity [89], which is part of the clinical picture of sarcopenia. According to the position of the European Working Group on Sarcopenia in the Elderly 2 (EWGSOP2), sarcopenia is a disease of the muscles characterised by reduced muscle strength and function, as well as low mass [16]. Although muscle weakness affects as many as 70% of SC patients, the incidence of sarcopenia in patients previously exposed to endogenous excess cortisol is currently unknown. On the other hand, EWGSOP2 has developed a comprehensive clinical algorithm to diagnose, confirm, and classify the severity of sarcopenia, an important element of which is the assessment of ALM and ALMI determined by the DXA method [16,78]. In combination with the previously described assessment of central obesity, the use of a whole-body DXA scan allows for a biplane clinical characterisation (central obesity and decreased skeletal muscle mass) of a patient with CS in a single examination.

Martel-Duguech et al. confirmed the usefulness of DXA testing for detecting sarcopenia in CS patients in long-term remission. Using EWGOSP criteria, including the ALMI analysis, the authors found sarcopenia in as many as 19% of the CS patients in long-term remission examined, which was higher than the control group matched for age, body mass index, menopausal status, and level of physical activity. The authors also noted that the demonstrated incidence of sarcopenia in the study group was similar to the incidence reported in the literature in the elderly (over 70 years of age (1–29%), as well as in the patients with type 2 diabetes (7.5–19.5%) [78].

Ragnarsson et al. used DXA in a cross-sectional case-control study to assess the impact of long-term outcomes in patients with Cushing’s disease (CD) and cortisol-producing adenomas on body composition. The researchers showed that the patients with CS in remission had a decreased percentage of ASMM, while they did not differ significantly in LBM with a matched control group [75].

Full-blown sarcopenia is common in CS patients, even in long-term remission. It seems that clinically reported muscle weakness, especially in the lower extremities, in CS may be due, among other things, to the increased infiltration of intramuscular fat observed during chronic exposure to excess cortisol [78,90,91]. ALMI is an important biomarker that allows clinicians to identify patients with sarcopenia; however, for this reason, its clinical relevance in CS may be diminished. However, the source data provided are insufficient for a clear position because data on this subject are scarce and only fragmentary data are available, as evidenced by the few sources included in this review.

#### 3.1.3. Bone Tissue Application

High rates of bone loss and fractures have been reported in CS through the deleterious effects of endogenous and exogenous hypercortisolism on bone metabolism. It is estimated that the prevalence of osteoporosis in CS patients is 40–70%, and osteopenia is as high as 80–85%. Source data indicate that fractures caused by hypercortisolaemia occur as early as the first year after the disease. In contrast, an improvement in bone densitometry is usually observed after two years of remission [92,93]. Therefore, the measurement of bone mineral density (BMD) by DXA is used as the primary surrogate of bone strength for diagnosing osteoporosis clinically in CS.

An example of the above is the study by Apaydın and Yavuz in which the BMD index was used to show a 40.7% incidence of osteopenia and a 16.2% incidence of osteoporosis in the group of CS patients examined. The authors also showed that the patients with CS had statistically significantly lower BMD and Z scores, both at the lumbar spine and femoral neck, compared to the control group. Lumbar spine BMD was found to be an independent predictor of vertebral fractures. Despite demonstrating statistically higher femoral neck BMD in patients in remission than in the patients with active CS, there was no statistically significant increase in BMD at a 2-year follow-up [56].

Guo et al. confirmed significantly lower lumbar, femoral, and whole-body BMD in the CD and adrenal-dependent Cushing’s syndrome (ACS) groups than in the control group. They found significantly lower lumbar spine BMD in the ACS group than in the CD group (*p* < 0.05). The concentrations of bone found showed significantly stronger suppression of bone formation and bone resorption in ACS patients than in CD patients, as confirmed by lower lumbar BMD in ACS patients than in CD subjects. The authors found a significant correlation between ACTH levels and lumbar BMD in CD patients, concluding that ACTH may have a protective effect on lumbar BMD. Based on the results, the researchers concluded that BMD testing should be a priority in fracture prevention, as it enables the early diagnosis of bone loss and allows for rapid prevention [79].

Since earlier studies have shown that changes in BMF (bone marrow fat) volume correlate with decreased BMD and a history of low-energy fractures in patients with osteoporosis or ageing, Maurice et al. used DXA to determine the BMD index. The BMD value served as one of the markers reflecting the biological and clinical effects of hypercortisolism and its consequences on bone metabolism. The authors summarised the results of BMF obtained by MRI with 1H-spectroscopy (1H-MRS). The authors confirmed a negative correlation between femoral BMF and BMD at the same site and a similar association between L3 BMF and total body BMD. Additionally, multiple regression analysis showed that femoral BMF was negatively associated with BMD and BMF content was negatively related to total body BMD [80].

In the study by Ragnarsson et al., there was no difference in BMD (total BMD and lumbar spine and femoral neck BMD) between the patients with CS in remission and the matched control group. This may have been influenced by 13 CS patients receiving osteoporosis treatment. The results obtained by the DXA method were used for genetic analyses, which allowed for the determination that GC dependency and the common NR3C1 Bcl1 polymorphism in the GC receptor gene are independently associated with reduced BMD in patients in remission [75].

The harmful effects of GC on bone remodelling are mainly realised through a marked decrease in bone formation with slightly increased or unchanged bone resorption markers [94]. Active hypercortisolism primarily affects cortical microarchitecture, which might explain the relatively subtle decreases observed in BMD. Although BMD usable from DXA is considered the gold standard for assessing bone tissue, it has some limitations affecting the ability to estimate bone strength and fracture risk. First, DXA does not measure true volumetric BMD and cannot distinguish between cortical and trabecular bone compartments, which may contribute differently to bone strength and fracture resistance. Some CS patients experience bone fractures and fractures despite having a low or even normal BMD value. Consequently, non-invasive methods to assess bone microarchitecture and bone quality, rather than just BMD and bone mineral content, are clearly desirable in these patients [81].

Therefore, Dos Santos et al. expanded the assessment of bone density by BMD DXA to include microarchitecture assessment by HR-pQCT in patients with endogenous Cushing’s syndrome. HR-pQCT detected lower cortical thickness, lower cortical area, and lower total and cortical density in the active CS group [82].

Vinolas et al. also point out that symptomatic fracture and BMD scores may be insufficient for estimating the presence of osteoporosis and osteopenia and should be expanded to assess bone microarchitecture. For this purpose, the authors propose to use trabecular bone score (TBS) as less invasive, associated with a lower radiation dose and more readily available (during DXA examination) compared to, for example, histomorphometric bone analysis and magnetic resonance tomography systems. The authors note that TBS is more frequently altered than BMD in overt CS and MACE (mild autonomic cortisol release) and is associated with fragility fractures and the severity of hypercortisolaemia. According to the authors, TBS is a valuable tool to complement the sub-baseline BMD score, allowing for the additional assessment of the extent of bone damage. Advantages of TBS include the relatively low cost of the test and the fact that it is derived from DXA measurements [83]. 

Ferraù et al. evaluated the association between BMF, as assessed by MRS, and morphometric VFs in patients with active endogenous CS. The authors provided the first evidence of an association between high bone marrow fat (BMF), low BMD, and a high prevalence of VFs in patients with CS. Although DXA does not capture abnormalities in bone microstructure induced by glucocorticoid excess, it allows the evaluation of morphometric vertebral fractures (VFs) in patients with CS. The study confirms the usefulness of the DXA method in evaluating skeletal fragility in Cushing’s syndrome (CS) [84].

Zhanna E. Belaya et al. looked for predictors of fractures in young patients with active endogenous Cushing’s syndrome. They showed a 44.5% frequency of osteoporotic fractures in the study group, with a predominance of vertebral compression fractures. The researchers found that CS patients are at risk of multiple fractures and that the risk of repeat fractures increases with the number of previous fractures. These authors noted that the patients with CS typically have low TBS (mean 1.107) and low reduced levels of bone formation markers, but low BMD loss. These results support the theory that the main detrimental impact of GC on bone may be structural rather than quantitative (bone mass, bone mineral density). In this young cohort of patients with active hypercorticism, BMD and TBS were not predictive of fracture. However, in the opinion of the authors, the best predictor of fractures in this group was the degree of the severity of hypercorticism, as measured by 24 h UFC levels, outweighing all other factors, including patient sex, age, BMI, BMD, TBS, and the degree of the suppression of bone formation markers [81].

With the DXA method, Trementino et al. found a 56% incidence of bone demineralisation (BMD) and a 25% incidence of fractures (VFA) in the patient group under study. In the patients with CD and ACS, they found a similar prevalence of osteoporosis and fractures. The prevalence of fractures was identical in post-menopausal and fertile women with CS. They found no significant differences in the prevalence of bone metabolism complications in men compared with women with CS. The researchers found that the degree of hypercortisolism affected both spinal bone loss (BMD values) and vertebral fractures (VFA). The authors confirmed that fractures were more likely to occur in patients with demineralisation of the bone. Z-scores at the lumbar spine and femoral neck were significantly lower in patients with CS fractures. The study found no association between the BclI and A3669G GR polymorphisms and BMD, T-score, and Z-score at the lumbar spine and femur, which are biochemical markers of bone resorption and bone apposition, in patients with overt CS. Based on the results, the researchers found that GR polymorphisms were not to be affected by the bone complications related to cortisol excess in patients with CS [25].

In the clinical use of BMD for diagnosis, osteoporosis monitoring and fracture risk prediction, one must also consider the effect of scanning artefacts on the outcome [95]. Scanning artefacts include, for example, the presence of osteophytes on bone and calcifications in the aorta, which can result in higher BMD values. Moreover, the heterogeneity of fat distribution can significantly affect the precision of the BMD result [96]. For example, BMD precision errors have been shown to positively correlate with increased BMI, heterogeneity, and the thickness of adipose tissue [97,98,99]. Abdominal adiposity, in particular, has been found to distort clinical BMD measurements and may affect osteoporosis status assessed by DXA, particularly at the lumbar spine but not at the hip [99]. The lower risk of precision error is due to the anatomy of the hip joint, where there are almost no large structures in the radiation path that can cause noise (such as abdominal fat or aortic calcification, which occur in the lumbar region) [100]. According to the authors, this is particularly relevant in individuals with Cushing’s syndrome, who typically have central obesity. This may also be the reason for the inconclusive findings regarding the diagnosis and severity of osteoporosis in these patients when using the lumbar spine BMD analysis alone. 

## 4. Conclusions

This systematic review focuses on the current applications and selected technical details of DXA testing in Cushing’s syndrome. DXA testing is undeniably an important tool in the clinical evaluation of Cushing’s syndrome. Indices of central obesity determined from the whole-body scan allow for assessing and monitoring changes in the content and distribution of adipose tissue both in the active and remission periods of Cushing’s syndrome. The lean mass analysis in assessing sarcopenia is unreliable in patients with CS due to the limitation of DXA in distinguishing muscle mass from intramuscular fat. Of the many indices obtained by DXA, BMD and VFA analyses are the most clinically relevant for CS patients. However, when using them clinically, it is important to keep in mind the high risk of precision errors in BMD estimated from the lumbar spine. 

## Figures and Tables

**Table 1:** Clinically relevant indicators from DXA.

Tissue	Type of Scan	Indicator	Formula	Unit	Clinical Application	Recommendations	Cut-Off Points	Refs.
Lean	Whole body	LMI	$\mathrm{LMI}=\frac{\mathrm{total}\;\mathrm{LM}}{{height}^{2}}$	kg/m^2^	Indices for the diagnosis of “low muscle mass” in sarcopenia and as a biomarker in the identification of undernutrition status			
		ALM	ALM = arms LM + legs LM	kg		EWGSOP2	♂ < 20 kg ♀ < 15 kg	[16]
		ALMI	$\mathrm{ALMI}=\frac{ALM}{{height}^{2}}$	kg/m^2^			♂ < 7.0 kg/m^2^ ♀ < 6.0 kg/m^2^	[16]
Adipose	Whole body	FMI	$\mathrm{FMI}=\frac{total\;FM}{{height}^{2}}$	kg/ m^2^	Proposed for the diagnosis of obesity	NHANES	Normal: ♀ = 5–9, ♂ =3–6 Overweight: ♀ = 9–13, ♂ = 6–9 Obesity class I: ♀ = 13–17, ♂ = 9–12 Obesity class II: ♀ = 17–21, ♂ = 12–15 Obesity class III: ♀ > 21, ♂ > 15	[45]
		AG	$\mathrm{AG}=\frac{Android\;ROI}{Gynoid\;ROI}$	n.a	Cardiovascular risk factors: dyslipidemia, insulin resistance, impaired glucose tolerance, hypercholesterolemia, hypertriglyceridemia, hypertension	n.a	AG > 1	[46,62]
		VAT	n.a	cm^2^; cm^3^; g	Metabolic and cardiovascular risk factors		Men ≥1025.0 cm^3^ (1086.0 g) is associated with the risk factors of MetS	[63]
							Women >100 cm^2^—metabolic risk >160 cm^2^—high metabolic risk	[64]
Bone	Whole body	BMD	$\mathrm{BMD}=\frac{bone\;mass}{bone\;area}$	g/cm^2^	Osteoporosis		Diagnosis in post-menopausal women and men aged 65 and older BMD T-score: normal: ≥−1 osteopenia: −1 to −2.5; osteoporosis: ≤−2.5 Diagnosis in premenopausal women, young men, children BMD Z-score * <2.0—low bone mass	
	Proximal femur					WHO ISCD *		[23]
	Lumbar spine							
	Lumbar spine	VFA	The fracture-grading method devised by Genant	n.a	Vertebral fractures	ISCD, AACCE, ACE	n.a	[28,65]
	Lumbar spine	TBS	The analysed grey-level variations in the 2D DXA images.	Trabeculogram, TBS values	Vertebral fractures	ISCD	Range 0.5 to 2.0, >1.35—normal 1.35—partial damage <1.20 serious damage	[66]

**Legend:** LMI—lean mass index; ALM—appendicular lean mass; ALMI—appendicular lean mass index; FMI—fat mass index; AG—android–gynoid ratio; VAT—visceral fat; BMD—bone mineral density; VFA—vertebral fracture assessment; TBS—trabecular bone score; NHANES—National Health and Nutrition Examination Survey; ISCD—The International Society for Clinical Densitometry; SFR—French Society for Rheumatology; GRIO—Osteoporosis Research and Information Group; AACCE—American Association of Clinical Endocrinologists; ACE—American College of Endocrinology; *—ISCD recommendation for BMD Z-score.

**Table 2:** Summary of found articles by adopted criteria.

Type of Tissue	Ref.	The Aim of the Study is Directly Connected with DXA	Sample (♀/♂)	Type of Scan/Indices	Densytometr Type
Fat tissue	[73]	To prospectively evaluate body composition changes with DXA in patients with active hypercortisolism and during the remission phase, considering the different therapeutic plans.	*n* = 23 (19/4) Active CS: 46.6 ± 12.2 yrs. Complete follow-up from 2009 to 2016. Remission CS: 51 ± 13.4 yrs. Controls *n* = 25 (21/4) 47 ± 9 yrs.	Whole-body scan: Total body: FM (g), FM (%), Lean (g), Lean + BMC (g) Trunk: FM (g), FM (%), Lean (g), Lean + BMC (g) R1 (the box was manually defined as DXA subregion 4 cm, slice at the top of the iliac crest): FM (g), FM (%), Lean (g), Lean + BMC (g)	Discovery W Hologic QDR 4500 C densitometer (Hologic Inc., Waltham, MA, USA)
	[74]	To investigate the adipose tissue distribution, adipocytokine profiles, and metabolic risk profiles of patients in long-term remission of CS.	*n* = 116 (92/24) CS in remission *n* = 58 (46/12) 50.8 ± 12.3 yrs. HC *n* = 58 (46/12) 51.2 ± 12.4 yrs.	Whole-body scan: FM (%), LBM (%), Trunk FM (%), Extremity FM (%), Leg FM (%), Trunk: leg, Trunk: extremities	Hologic QDR 4500 densitometer (Hologic, Bedford, Zaventem, Belgium)
	[75]	To study body composition and BMD in patients with CS in long-term remission.	*n* = 100 (100/0) CS in remission *n* = 50 (50/0) 53 ± 14 yrs. HC *n* = 50 (50/0) age-matched	Scan of: lumbar spine, femur neck; BMD (g/cm^2^) Whole-body scan: LBM (kg), LBM (% of body weight) FM (kg), FM (% of body weight) abdominal FM (kg), abdominal FM (% of body weight) ALM (kg), ALM (% of body weight)	Lunar DPX-L, 12 Lunar Corporation, Madison, WI, USA).
	[76]	To develop a set of normative standards, reference ranges with the determination of the cut-off points (CP) values of DXA indices of central abdominal obesity (COIs) as a ratio of android (A) to gynoid (G) fat and tissue mass and their percentages that best differentiate CS and obese women (O) and confirm central abdominal obesity and to determine their normal CP values that best differentiate group C from CS, O1, and O2 and exclude abdominal obesity.	*n* = 72 (72/0) CS *n* = 18 (18/0) 43.58 ± 13.58 yrs. O1 *n* = 18 (18/0) 40.4 ± 12.05 yrs. O2 *n* = 18 (18/0) 45 ± 8 yrs. HW *n* = 18 (18/0) 40.09 ± 12.72 yrs.	Whole-body scan: COI1 = $\frac{At}{Gt}$, COI2 = $\frac{Af}{Gf}$, COI3 = $\frac{At\%}{Gt\%}$, COI4 = $\frac{Af\%}{Gf\%}$	System Lunar DPX-NT, enCore
	[77]	To develop CP values of new DXA indices of central abdominal obesity as ratios of android and trunk to legs, as well as trunk and legs to total fat and tissue mass and their percentages that best differentiate CS and O1 and confirm central abdominal obesity (m) and to determine their normal (n) CP values that best differentiate group C from CS, O1, and O2 and exclude abdominal obesity.		Whole-body scan: A/L ratios: A/L-t, A/L-f, A/L-t%, A/L-f%, Tr/L ratios: Tr/L-t, Tr/L-f, Tr/L-f%, Tr/L-f% Tr/To: Tr/To-t, Tr/To- f, Tr-f%, Tr-f%, L/To ratios: L/To-t, L/To-f, L/To-t%, L-Tof%	System Lunar DPX-NT, enCore
Lean tissue	[78]	To assess the prevalence of sarcopenia in CS patients in long-term remission using the EWGSOP2 criteria.	*n* = 72 (72/0) CS in remission 36 (36/0) median age 51 (45.2–60) yrs. Controls *n* = 36 (36/0) age-matched	Whole-body scan: ALM (kg) ALMI (kg/m^2^)	Hologic Discovery W, Software Apex Version 13.4
Bone tissue	[56]	(1) To determine the frequency of non-traumatic vertebral fractures in patients with CS and the factors associated with vertebral fractures. (2) To assess bone mineral density in patients with CS.	*n* = 242 (188/54) Active CS *n* = 135 (111/24) HC *n* = 107 (77/30) age-matched	Scan of: lumbar spine, femoral neck; BMD (g/cm^2^), T-score, Z-score	Lunar DPX-L
	[79]	(1) To characterise BMD and bone metabolism in patients with ACTH-dependent or ACTH-independent hypercortisolism versus healthy controls. (2) To analyse the effects of ACTH on BMD in patients with ACS and CD.	*n* = 73 (57/16) ACS *n* = 21 (19/2) 35 ± 11.53 yrs. CD *n* = 34 (25/9) 36.63 ± 11.22 yrs. HC *n* = 18 (13/5) 38.36 ± 14.19 yrs.	Scan of: lumbar spine, femoral neck, whole body, BMD (g/cm^2^), T-score, Z-score	Prodigy-GE densitometer (GE Healthcare, Chicago, IL, USA)
	[80]	To evaluate bone marrow fat by 1H-MRS in patients in remission (at least two years) vs active ACTH-dependent CS in comparison with age- and sex-matched controls.	*n* = 51 (38/13) Active CS *n* = 17 (12/5) 51.6 ± 3.4 yrs. Remission CS *n* = 17 (13/4) 48.3 ± 3.0 yrs. HC *n* = 17 (13/4) 50.3 ± 2.1 yrs.	Scan of: lumbar spine, femoral neck; BMD (g/cm^2^), T-score	GE Lunar iDXA (Healthcare)
	[81]	To identify risk factors for fracture in patients with endogenous CS and to evaluate the value of bone microarchitecture as assessed by TBS in this cohort of patients.	CS *n* = 182 (149/33) Patients with fractures *n* = 81 (59/22) 39.3 ± 12.9 yrs. Patients without fractures, *n* = 101 (90/11) 36.6 ± 12.4 yrs.	Scan of: lumbar spine, femoral neck, hip mean spine TBS, mean spine TBS Z- score, mean spine TBS BMD (g/cm^2^), femoral neck BMD (g/cm^2^), femoral neck Z-score, total hip BMD (g/cm^2^), total hip BMD Z-score	Lunar Prodigy (GEHC Lunar, Madison, Madison, WI, USA)
	[82]	To investigate alterations in BMD (evaluated by DXA and HR-pQCT) and bone microarchitecture (evaluated by HR-pQCT) in a cohort of patients with active endogenous CS.	*n* = 81 (68/13) Active CS (24/6) 38.1 ± 14 yrs. HC (44/7) 36.1 ± 8.3 yrs.	Scan of: lumbar spine, femoral neck, total femur, radius; BMD (g/cm^2^), T-score, Z-score	GE Lunar Prodigy Advance; (GE Healthcare Madison, Madison, WI, USA)
	[83]	To compare lumbar spine BMD and TBS in a large cohort of cases of overt CS and MACE of various etiologies and to assess the evolution of BMD and TBS in the subset of patients after remission of overt CS.	*n* = 110 (89/21) CS *n* = 53 (42/11) 49.9 ± 12.8 yrs. MACE *n* = 39 (34/5) 57.8 ± 9.3 yrs. patients with non-secreting adrenal incidentalomas *n* = 18 (13/5) 59.2 ± 9.1 yrs.	Lumbar spine scan BMD (g/cm^2^), T-scores; TBS	Lunar iDXA, GE, Madison, USA; TBS insight v2.1 software (Medimaps, Mérignac, France).
	[84]	To evaluate the association between bone marrow fat (BNF), as assessed by magnetic resonance spectroscopy (MRS), and morphometric vertebral fractures (VFs) in patients with active endogenous CS.	*n* = 35 (26/9) Active endogenous CS *n* = 20 (15/5) 44 ± 13 yrs. HC *n*= 15 (11/4) age 43 ± 12 yrs.	Lumbar spine scan BMD (g/cm^2^) A quantitative morphometric; six points were marked on each vertebral body to describe: anterior (Ha), middle (Hm), and posterior (Hp) vertebral heights; Height ratios (Ha/Hp, Hm/Hp, Hp/Hp of the above vertebrae, Hp/Hp of the below vertebrae) were calculated for each vertebra from L1 to L4;	Hologic Discovery W
	[25]	(1) To evaluate the prevalence of bone complications (bone demineralisation, vertebral and peripheral fractures) in patients with CS. (2) To investigate the role of gender, disease aetiology, duration, and degree of hypercortisolism, as well as the impact of GR gene polymorphisms, on the development of these complications.	*n* = 52 (43/9) CD *n* = 38 (32/6) 43.58 ± 13.23 yrs. ACS *n* = 14 (11/3) 48.50 ± 1 5.05 yrs.	DXA morphometry; Scan of: lumbar spine, femoral neck, total femur; BMD (g/cm^2^), T-score, Z-score	Lunar Prodigy^®^ DXA (GE Healthcare, Madison, WI, USA). enCore 2007 version 11.4.

**Legend:** CS—Cushing’s syndrome; O1—obese women not different according to their age and BMI from CS; O2—obese women with BMI of 35 ± 1.2 kg; HW—healthy women; A—android regions; G—gynoid regions; COI—central obesity indices; t—tissue mass; f—fat mass; L—legs; Tr—trunk; To—total; m—abdominal obesity; n—normal body composition; CD—Cushing’s disease; ACS—adrenal Cushing’s syndrome; HC—healthy controls; BMD—bone mineral density; FM—fat mass; ALM—appendicular lean mass; ALMI—appendicular lean mass index; HR-pQCT—high-resolution peripheral quantitative computed tomography.

## Data Availability

Not applicable.
